# Applying multilayer analysis to morphological, structural, and functional brain networks to identify relevant dysfunction patterns

**DOI:** 10.1162/netn_a_00258

**Published:** 2022-07-01

**Authors:** Jordi Casas-Roma, Eloy Martinez-Heras, Albert Solé-Ribalta, Elisabeth Solana, Elisabet Lopez-Soley, Francesc Vivó, Marcos Diaz-Hurtado, Salut Alba-Arbalat, Maria Sepulveda, Yolanda Blanco, Albert Saiz, Javier Borge-Holthoefer, Sara Llufriu, Ferran Prados

**Affiliations:** e-Health Center, Universitat Oberta de Catalunya, Barcelona, Spain; Center of Neuroimmunology, Laboratory of Advanced Imaging in Neuroimmunological Diseases (ImaginEM), Hospital Clínic de Barcelona, Institut d’Investigacions Biomèdiques August Pi i Sunyer (IDIBAPS), Universitat de Barcelona, Barcelona, Spain; IN3, Universitat Oberta de Catalunya, Barcelona, Spain; Centre for Medical Image Computing, Department of Medical Physics and Biomedical Engineering, University College London, London, United Kingdom; Queen Square MS Centre, Department of Neuroinflammation, UCL Institute of Neurology, Faculty of Brain Sciences, University College London, London, United Kingdom

**Keywords:** Structural connectivity, Functional connectivity, Gray matter networks, Multiple sclerosis, Multilayer

## Abstract

In recent years, research on network analysis applied to MRI data has advanced significantly. However, the majority of the studies are limited to single networks obtained from resting-state fMRI, diffusion MRI, or gray matter probability maps derived from T1 images. Although a limited number of previous studies have combined two of these networks, none have introduced a framework to combine morphological, structural, and functional brain connectivity networks. The aim of this study was to combine the morphological, structural, and functional information, thus defining a new multilayer network perspective. This has proved advantageous when jointly analyzing multiple types of relational data from the same objects simultaneously using graph- mining techniques. The main contribution of this research is the design, development, and validation of a framework that merges these three layers of information into one multilayer network that links and relates the integrity of white matter connections with gray matter probability maps and resting-state fMRI. To validate our framework, several metrics from graph theory are expanded and adapted to our specific domain characteristics. This proof of concept was applied to a cohort of people with multiple sclerosis, and results show that several brain regions with a synchronized connectivity deterioration could be identified.

## Author Summary

This study presents the design, development, and validation of a framework that merges morphological, structural, and functional brain connectivity networks into one multilayer network. To validate our framework, several metrics from graph theory are expanded and adapted to our specific domain characteristics. This proof of concept was applied to a cohort of people with multiple sclerosis, and results show that some brain regions with a synchronized connectivity deterioration could be identified.

## INTRODUCTION

In the field of neuroscience, studying brain networks in the context of both health and disease is common and plays a critical role in shedding light on the brain mechanisms driving cognitive processes such as learning or reasoning, as well as addressing brain damage (Bassett & Sporns, 2017). Recent advances in magnetic resonance imaging (MRI) have facilitated the study of brain connectivity structures and functions and provide a comprehensive understanding of aspects of brain connectivity behavior and organization (Bennett & Rypma, 2013; Groppa et al., 2021). Graph theoretical analysis enables us to model complex network systems with comprehensive indices related to the integration, segregation, and propagation of information inside the brain system (Sporns, 2013). However, most studies focus on only the topological characteristics of brain structural or functional connectivity metrics without considering the interactions between them. This approach limits knowledge on how the brain works or how it responds to damage. It is our hypothesis that integrating information on structural connectivity based on fractional anisotropy (FA)-weighted connectivity, gray matter (GM) morphological association networks, and functional connections studied through resting-state functional connectivity can provide further insights and new knowledge on brain organization.

This integration of data could be carried out taking multiple approaches used in previous studies. For example, some indirect methods analyze each network separately using the same graph theory metrics, and then merge the quantitative brain network properties using advanced statistical methods (Shu et al., 2016). Other indirect methods apply machine learning techniques to extract different patterns or obtain groups from the networks analyzed (Zitnik et al., 2019). A further method uses more advanced techniques such as deep learning methods and graph neural networks (GNN; Ma & Tang, 2021) to directly analyze all the networks simultaneously. Yet another strategy, known as multilayer or multiplex networks, integrates all the networks into a single higher dimensional graph, and with the information from all the networks compiled, can perform a direct data analysis.

In terms of flexibility, this method can integrate information from structural and functional graph theoretical analysis into a multilayer network framework, and extract the multilayer organization of human brain connectivity in a neurological disease context, such as multiple sclerosis (MS; Chard et al., 2021). MS is a chronic, inflammatory, demyelinating, and neurodegenerative disease of the central nervous system characterized by widespread damage leading to disruption of large- and short-scale structural and functional connectivity, which leads to clinical alterations (Fleischer et al., 2019; Rocca et al., 2015). Local and global implications of damage on networked systems, such as our brain, have been studied from many angles using complex networks (Newman, 2018). Thus, network theory approaches have been widely applied in the field of neuroscience to study both structural and functional connectivity and explore its relationship with cognitive function (Llufriu et al., 2017; Pagani et al., 2020; Rocca et al., 2016). However, in this context, single network analysis is limited to only one feature, and hence, does not fully describe the complexity of brain mechanisms after damage.

The multilayer network approach (Kivelä et al., 2014; Boccaletti et al., 2014), on the other hand, enables one to incorporate different types of relational information between brain regions, and additionally to encode their cross relationships. This generic modeling framework can be exploited in different ways to study the brain in its different states (De Domenico, 2017; Muldoon & Bassett, 2016), such as frequency-based decompositions (Buldú & Porter, 2018; Guillon et al., 2017), time-varying networks (Betzel et al., 2019; Pedersen et al., 2018; Ting et al., 2021), or structural and functional decomposition (Battiston et al., 2017; Battiston et al., 2018), although with drawbacks and challenges (Mandke et al., 2018). Within this last multilayer framework, the brain is divided into different regions that are represented by network nodes with a one-to-one correspondence between the nodes in the different layers that represent different brain modality (e.g., EEG, fMRI, dMRI). This provides, to date, the most robust approach to integrate different types of brain networks into a single framework, rather than simply joining and combining information obtained considering the layers independently, or extracting them from the aggregated network.

Multilayer networks have been used in the past to study brain function at many levels—microscale (De Domenico et al., 2016), mesoscale (Battiston et al., 2017; Battiston et al., 2018; Crofts et al., 2016; Ting et al., 2021), and macroscale (Buldú & Porter, 2018; Cociu et al., 2018; Pedersen et al., 2018; Suárez et al., 2020)—and have been applied to the analysis of different types of disorders (Gifford et al., 2020; Guillon et al., 2017; Guillon et al., 2019; Liu et al., 2017) or normal brain functioning, or simply to understand the brain’s organization (Betzel et al., 2019; De Domenico, 2017; Muldoon & Bassett, 2016). Interestingly, each different disorder requires different adaptations of the general multilayer framework: definition of layers and interlinks, which may take advantage of the information that can be obtained from the different layers individually (Liu et al., 2017).

This study presents the conceptual framework that contributes to the state of the art from two points. We first explore and extend the brain multilayer framework to habilitate the joint analysis of morphological, structural, and functional networks, which to date was limited to only structural and functional networks. From there, we expand several metrics from network theory to analyze the proposed multilayer scheme. Finally, the multilayer framework is adapted to study a cohort of people with MS as a proof of concept, and we detect several brain regions with a synchronized connectivity deterioration.

## MATERIAL AND METHODS

### Participants

This study used data on patients with relapsing-remitting MS aged 18–65 years, and consecutively recruited at the MS Unit at Hospital Clínic de Barcelona. The cohort was composed of 125 subjects diagnosed with MS according to the 2010 McDonald criteria (Polman et al., 2011) and 45 healthy volunteers (HV) without relapses in the last 3 months or a previous history of psychiatric diseases. Physical disability was evaluated using the Expanded Disability Status Scale (EDSS). The Ethics Committee of the Hospital Clínic de Barcelona approved the study, and all participants signed an informed consent.

### Magnetic Resonance Acquisition Details

MRI acquisition protocols were acquired on a 3T Magnetom Trio scanner (SIEMENS, Erlanger, Germany) using a 32-channel phased-array head coil. In part of the cohort (*n* = 87 participants), the high-resolution three-dimensional magnetization-prepared rapid acquisition with gradient echo (3D-MPRAGE) was acquired with TR = 1,800 ms; TE = 3.01 ms; TI = 900 ms; 240 sagittal slices with 0.94 mm isotropic voxel size; and a 256 × 256 matrix size; and the three-dimensional fluid-attenuated inversion recovery (3D-T2 FLAIR) with TR = 5,000 ms; TE = 304 ms; TI = 1,800 ms; 192 sagittal slices with 0.94 mm isotropic voxel size; and a 256 × 256 matrix size. The diffusion-weighted imaging (DWI) had a TR = 14,800 ms; TE = 103 ms; 100 contiguous axial slices; 1.5 mm isotropic voxel size; a 154 × 154 matrix size; b-value = 1,000 s/mm^2^; 60 diffusion encoding directions; and a single baseline image acquired at 0 s/mm^2^. The remaining participants (*n* = 83) had a 3D-structural image with TR = 1,970 ms; TE = 2.41 ms; TI = 1,050 ms; 208 sagittal slices with 0.9 mm isotropic voxel size; and a 256 × 256 matrix size; and the 3D-T2 FLAIR with TR = 5,000 ms; TE = 393 ms; TI = 1,800 ms; 208 sagittal slices with 0.9 mm isotropic voxel size; and a 256 × 256 matrix size. The DWI acquisition protocol was as follows: TR = 12,600 ms; TE = 112 ms; 80 contiguous axial slices; 2 mm isotropic voxel size; a 120 × 120 matrix size; b-value = 1,500 s/mm^2^; 70 diffusion encoding directions; and a single baseline image acquired at 0 s/mm^2^. In addition, field map images were generated for all participants and used to correct distortions caused by field inhomogeneities (TE 1 / TE 2 = 4.92/7.38 ms, with the same slice prescription, slice thickness, and field of view as the DWI sequence).

For 143 subjects (125 people with MS and 18 HV), the same resting-state functional MRI (rs-fMRI) protocol was acquired using BOLD EPI pulse sequence (fat saturation), with TR = 2,000 ms; TE = 19 ms; field of view = 220 mm; 40 contiguous axial slices with 1.7 × 1.7 × 3 mm voxel size; GRAPPA-factor of 4; and a total of 450 frames (TA = 15:14 min).

### Data Processing

#### Anatomical parcellation scheme.

White matter lesions were delineated with 3D-MPRAGE and 3D-FLAIR images using JIM7 software (https://www.xinapse.com/). Subsequently, white matter lesions were filled in 3D-MPRAGE with the intensity of the non-lesional neighboring voxels to improve MS patient registration and segmentation processing (Battaglini et al., 2012). Lesion-filled images were used to parcellate the cortex into 62 GM regions and 14 subcortical regions using Mindboggle software and FSL-FIRST packages, respectively (Klein et al., 2017; Patenaude et al., 2011). The anatomical cortical parcellation computed by Mindboogle was extracted from the Desikan-Killiany-Tourville atlas (Desikan et al., 2006). The nodes of the three brain networks constructed are the 76 brain regions depicted; thus, the same parcellation is used within each network.

#### Structural brain connectivity network.

The first step in constructing a structural connectivity matrix was to build a DWI preprocessing pipeline to fit the diffusion tensor imaging (DTI) model, an approach previously described and well established by Tournier et al. (2019). Major components of the pipeline included MP-PCA denoising, Gibbs ringing removal, eddy current and motion correction, geometrically unwarping procedure, and bias field correction. Once these corrections were made, the FA scalar map was computed from the DTI model using FSL’s DTIFIT (Basser et al., 1994). The structural connectivity matrices derived from FA-weighted indices were generated using the results from the high-order probabilistic streamline tractography between distinct cortical and subcortical GM areas (nodes) of the whole brain. To guarantee biologically plausible reconstructed streamlines, the connectome reconstruction process incorporated the anatomical constrained tractography (ACT) framework, from which a set of six million streamlines were selected, and postprocessing based on anatomical exclusion criteria (Llufriu et al., 2017; Martínez-Heras et al., 2015). The parcellation scheme (76 nodes) from the anatomical image was aligned to the FA map to determine which streamline connections needed to be selected between pairs of nodes to create the structural connectome. We defined the mean value of the FA metric along each connection to generate the FA-weighted adjacency matrix of the network, denoted by *A*^(*DTI*)^. The mean FA computed along the fiber pathway that connects each pair of brain regions enables the inclusion of the severity of the white matter damage at the macro- and microstructural levels (Llufriu et al., 2017). In order to minimize the presence of false connections in the networks, the analysis included only network links that were present in more than 60% of the 45 HV subjects. Finally, the FA measures for the structural network were corrected for age and gender effect using a regression model (Solana et al., 2019). The values of DTI connectivity matrices are in the range [0, 1].

#### Morphological gray matter brain network.

The GM morphological network is based on the similarity of GM morphological patterns according to the defined anatomical parcellation scheme (Tijms et al., 2012). To estimate this GM connectivity network, we used an automated pipeline, which involved four main steps: (a) re-slicing each individual’s native space GM segmentation to 2 mm isotropic voxel in MNI space to later define small regions of interest corresponding to 3 × 3 × 3 voxel cubes (6 × 6 × 6 mm^3^); (b) performing statistical similarity (Pearson’s correlation coefficient) between each pair of cubes of the GM mask; (c) applying an individualized threshold to ensure a similar percentage of spurious connections (5%) across all GM networks; and (d) averaging the remaining correlation values within each anatomical node in order to construct the final GM morphological network, its adjacency matrix being denoted by *A*^(*GM*)^, considering the defined parcellation scheme (76 × 76). The morphological networks obtained were corrected for the effects of age and gender using a regression model. The values of GM morphological matrices are in the range [0, 1].

#### Functional brain network.

Brain signal correlation/synchronization through resting-state functional connectivity (rs-fMRI) matrix was obtained following Chou et al. (2012). This includes several preprocessing steps including slice timing and motion correction, spatial normalization to a standard space, and band-pass filtering to retain frequencies between 0.001 and 0.08 Hz using FSL tools (Jenkinson et al., 2012). Finally, the defined parcellation was used to extract the average time series for each of the 76 brain regions, resulting in a functional connectivity network with adjacency matrix *A*^(*rsfMRI*)^. Note that the values of rs-fMRI matrices are in the range [−1, 1], indicating negative or positive correlation between nodes. However, we apply the absolute value in order to preserve only the strength of the relationship, to simplify and avoid relevant drawbacks when computing network descriptors related to distance or shortest paths. As with the other networks, age and gender effects were also corrected for functional connectivity networks using a regression model. The final values of rs-fMRI matrices are in the range [0, 1].

### Multilayer Brain Network Definition

A multilayer network is composed of different layers, each representing a single type of relationship between nodes within one layer. Nodes represent the same exact object in each of the different layers, and encode different types of relationships throughout their edges. In this type of network, we differentiate between intralayer links, which encode the single type of relationship the layer represents, and interlayer links, which encode how the different node perspectives (types of relationships) are related within the system.

**Figure 1.**
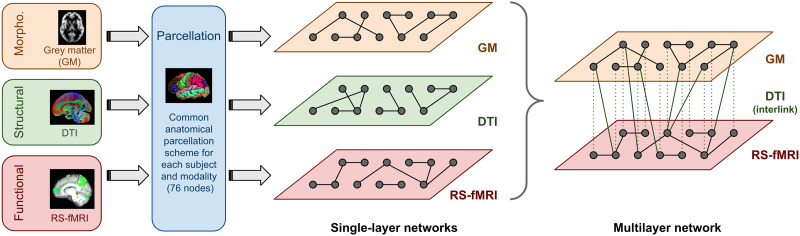
Multilayer network definition scheme using morphological, structural, and functional brain networks with the same underlying anatomical parcellation.

In our particular case, each subject has three single-layer networks representing GM morphology, DTI structural connectivity, and rs-fMRI functional activity, which we combine to create a multilayer network composed of two layers (Figure 1). We propose encoding relational data on GM tissue (GM, covariance in cortical thickness, and rs-fMRI, correlation of functional time series) within the layers of the multilayer object, and encoding white matter structural information (DTI) with interlayer links. This option was decided upon as DTI structural connectivity matrices represent white matter fiber pathway integrity between GM brain regions through a range of values between 0 (isotropic diffusivity) and 1 (anisotropic diffusivity; Llufriu et al., 2017).

**Figure 2.**
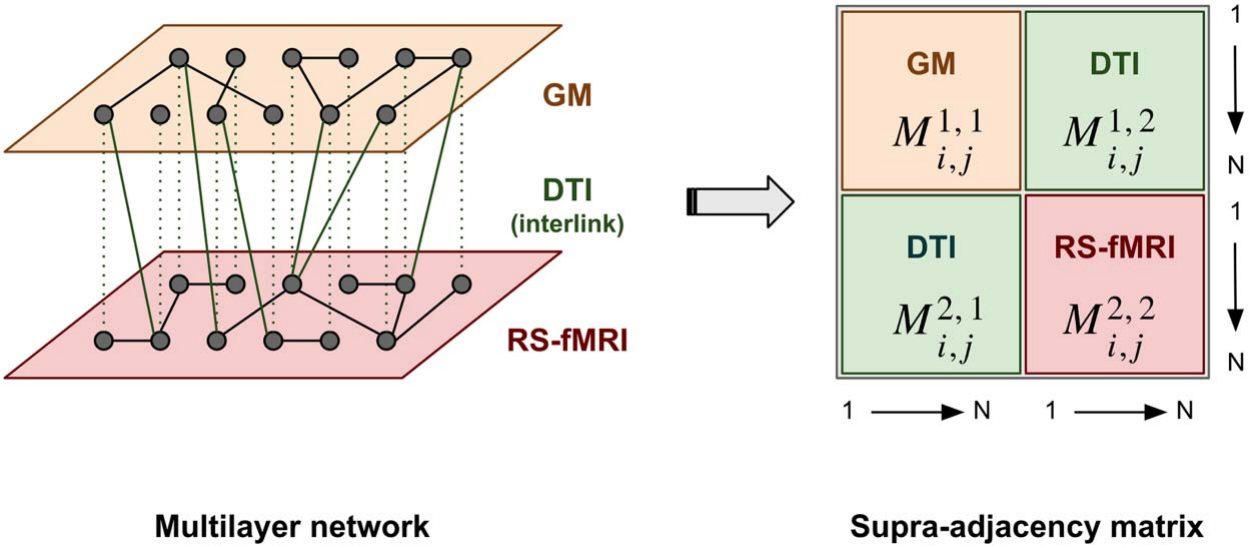
Multilayer adjacency matrix unfolded to a supra-adjacency matrix, interlinked by structural DTI connectivity.

Figure 1 shows the procedure used to assemble the multilayer object. Since the morphological GM, structural DTI, and rs-fMRI functional brain networks are already built on a common parcellation atlas, and a statistical correction for age and gender applied, the construction of the multilayer object is not problematic. The multilayer information is represented by a **multilayer adjacency matrix**, $M_{i,j}^{\alpha ,\beta }$, composed of four indices. Two indices are the layers, denoted by the Greek letters *α* and *β*, and two are the nodes in the layers, denoted by the Latin letters *i* and *j*. Following on from the single-layer networks defined in the previous sections, the multilayer adjacency matrix is defined as follows:

$$M_{i,j}^{1,1}=A_{i,j}^{\left(GM\right)}\forall i,j\in \left\{1,\ldots ,N\right\},$$

$$M_{i,j}^{2,2}=A_{i,j}^{\left(\text{rsfMRI}\right)}\forall i,j\in \left\{1,\ldots ,N\right\},$$

$$M_{i,j}^{1,2}=M_{i,j}^{2,1}=A_{i,j}^{\left(DTI\right)}\forall i,j\in \left\{1,\ldots ,N\right\},$$

where $M_{i,i}^{\alpha ,\beta }$ ≈ 0, since we assume maximum connectivity between the same brain parcels corresponding to different layers. The morphological GM network is encoded in the first layer of the multilayer object, and the rs-fMRI functional network in the second layer; therefore, as stated previously, interlayer links (i.e., indices *α* ≠ *β*) are defined as the DTI integrity between the different brain areas (GM anatomical regions) represented by the structural network. Figure 2 shows how this multilayer network unfolds into a supra-adjacency matrix (Kivelä et al., 2014). Throughout the paper, the multilayer adjacency matrix is used directly, $M_{i,j}^{\alpha ,\beta }$.

In relation to the multilayer adjacency matrix, an additional object of lower dimension is defined to show minimum distances in $M_{i,j}^{\alpha ,\beta }$. The **matrix of the minimum distances** (*D* ∈ *R*^*N*×*N*^) encodes the minimum distance between each pair of nodes in the multilayer network, independent of the path or layer used to connect the two nodes. Despite this being an approximation, this safely simplifies the multilayer object, as connectivity between replicas of the same node in the different layers is assumed to be maximum. This, therefore, minimizes the cost in the multilayer adjacency matrix, $M_{i,i}^{\alpha ,\beta }$ ≈ 0. Note that the information contained in this matrix is partial and derived from the multilayer network and, in general, it is not possible to reconstruct the original multilayer network from the minimum distance matrix. Formally, we obtain the minimum distance matrix:

$$D_{i,j}=\min\;M_{i,j}^{\alpha ,\beta }:\alpha ,\beta \in \left\{1,2\right\};i,j\in \left\{1,\ldots ,N\right\}.$$

As we have stated previously, all values of intra- and interlinks are encoded in the range [0, 1] because of the preprocessing steps that we have applied to obtain the single-layer adjacency matrix. However, we underline that different or alternative preprocessing steps could be implemented and integrated in the multilayer framework defined here, such as the mean or any other relationship between values (Dimitriadis et al., 2017; Yu et al., 2017).

### Graph Theoretical Analysis

Although several metrics for multilayer networks have been defined previously (De Domenico et al., 2013; Solé-Ribalta et al., 2014), some metrics may need to be adjusted when the particular domain characteristics are taken into account. Particularly, the complex network descriptors related to the information flow between nodes (i.e., regions of the brain) may be within the same layers and/or different layers. Therefore, we propose using well-known global and local measures to describe the multilayer brain network properties at both local and global levels. We formulate the following.

#### Strength.

Node strength is one of the most basic and widely used metrics to calculate the importance of any given node in a network, and is defined as the sum of weights of all edges connected to the node. According to this definition, high strength indicates a highly important node in the network. We define the strength of node *i*, denoted by *s*_*i*_, as the following:

$$s_{i}=\sum_{\alpha }s_{i}^{\alpha }\;\text{with}\;s_{i}^{\alpha }=\sum_{\beta ,j}M_{i,j}^{\alpha ,\beta }.$$

In a multilayer network we can define node strength at two levels: a local level, $s_{i}^{\alpha }$, which notes the importance of node *i* in layer *α*, without reducing it to the sum of the degrees of the individual layers (De Domenico et al., 2015); and a more general level that aggregates the overall structural importance of node *i* considering all layers, *s*_*i*_. Although $s_{i}^{\alpha }$ may be useful to quantify the local importance of node *i*, this research only focuses on the aggregated node strength *s*_*i*_.

#### Degree.

Node degree is one of the simplest centrality measures (often used in social network literature) to quantify the importance of a node in a network. It is defined as the number of edges connected to a specific node, and can be considered a binarization of strength. In a similar way, high degree values indicate higher importance of the multilayer node. We define the degree of node *i* as follows:

$$d_{i}=\sum_{\alpha ,\beta ,j}\Theta \left(M_{i,j}^{\alpha ,\beta }\right),$$

where *θ* is a function that returns 0 if the argument is equal to 0, and 1 otherwise. In other words, it returns 1 if an edge exists between node *i* in layer *α* and node *j* in layer *β*, and 0 otherwise. Note that, if required, one could also define the degree per node and layer, $d_{i}^{\alpha }$, as described above for strength.

#### Betweenness centrality.

A different concept of node importance is captured by betweenness centrality, which measures the extent to which a node lies within paths between node pairs (Freeman, 1977). In contrast to degree and strength, this node metric is obtained by considering the full network topology, and is related to the information flow between nodes (within the same layer or across different layers). The betweenness centrality of node *i* is set out in the following equation:

$$b_{i}=\frac{1}{n\left(n-1\right)}\sum_{o,d}\frac{\sigma_{o,d}\left(i\right)}{\sigma_{o,d}},$$

where *σ*_*o*,*d*_ is the number of the shortest paths from *o* to *d*, and *σ*_*o*,*d*_(*i*) is the number of the shortest paths from *o* to *d* that crosses node *i*. The betweenness centrality is a value in the range [0, 1], where 1 indicates the highest centrality (i.e., the node lies in all the shortest paths from all pairs of nodes) and 0 denotes a node that is not in any of the network’s shortest paths.

In our particular setup, we can efficiently compute the betweenness centrality using the matrix of minimum distances, *D*, which is directly derived from the multilayer network. By using *D*, the standard betweenness centrality algorithm (Brandes, 2001) can be applied. It is worth noting that our definition and computation of betweenness centrality is slightly different from the original one proposed for multilayer schemes (Solé-Ribalta et al., 2014).

#### Closeness centrality.

Closeness centrality evaluates the average distance from one node to all other accessible nodes in the network. Similar to the previous metric, this is a global network descriptor, and is related to the topological location of nodes within the network. The closeness centrality of node *i* is computed as the average distance between *i* and all other nodes in the network:

$$c_{i}={\left(\sum_{j\neq i}D_{i,j}\right)}^{-1},$$

where values close to 1 denote very high centrality and values close to 0 indicate very low centrality. It should be highlighted that under this metric definition, the more central a node is, the closer it is to all other nodes.

#### Local efficiency.

A network’s efficiency is a measure of its capacity to exchange information between nodes (Latora & Marchiori, 2001; Stanley et al., 2015). At a global level, efficiency is defined as the sum of the inverse of all pairwise distances between nodes and measures how well information is exchanged within the network. At the local level, it measures how well a node’s neighbors can exchange information between them, and it can be used to measure a network’s resistance to failure at a local level (Latora & Marchiori, 2001). In the context of brain networks, local and global efficiency have been linked to working memory (Stanley et al., 2015) and functional integration (Rubinov & Sporns, 2010). Formally, the local efficiency of node *i* is computed by the following equation:

$$LE_{i}=\frac{1}{d_{i}\left(d_{i}-1\right)}\sum_{j,k\in G_{i}}\frac{1}{D_{j,k}},$$

where *G*_*i*_ is the set containing the immediate neighborhood of node *i* (also called the ego network), but excluding the node itself, and *d*_*i*_ is the degree of node *i* as defined above. Analogous to the other measures, the local efficiency of node *i* is in the range [0, 1], where values close to 1 indicate maximum local efficiency in the network.

### Statistics

Clinical and demographic data were described through the mean and standard deviation for quantitative variables. Comparisons of global and local network descriptors between people with MS and HVs were performed with Student’s *t* tests, and the statistical significance was set at *p* < 0.05. Since the FA-weighted adjacency matrices could suffer from intersite variability because of the heterogeneity of both acquisition protocols, we harmonized the data using the ComBat model (Fortin et al., 2017; De Stefano et al., 2022). All analyses were performed using Python software (version 3.8.8) and the SciPy package (version 1.8.0).

### Data and Code Availability

The proposed method to create a multilayer network derived from FA-weighted adjacency matrix, GM morphological and functional brain networks, and the subsequently graph theoretical analysis were made publicly available by the authors in the following link: https://github.com/ADaS-Lab/Multilayer-MRI/ (Casas-Roma, 2022).

## RESULTS

**Figure 3.**
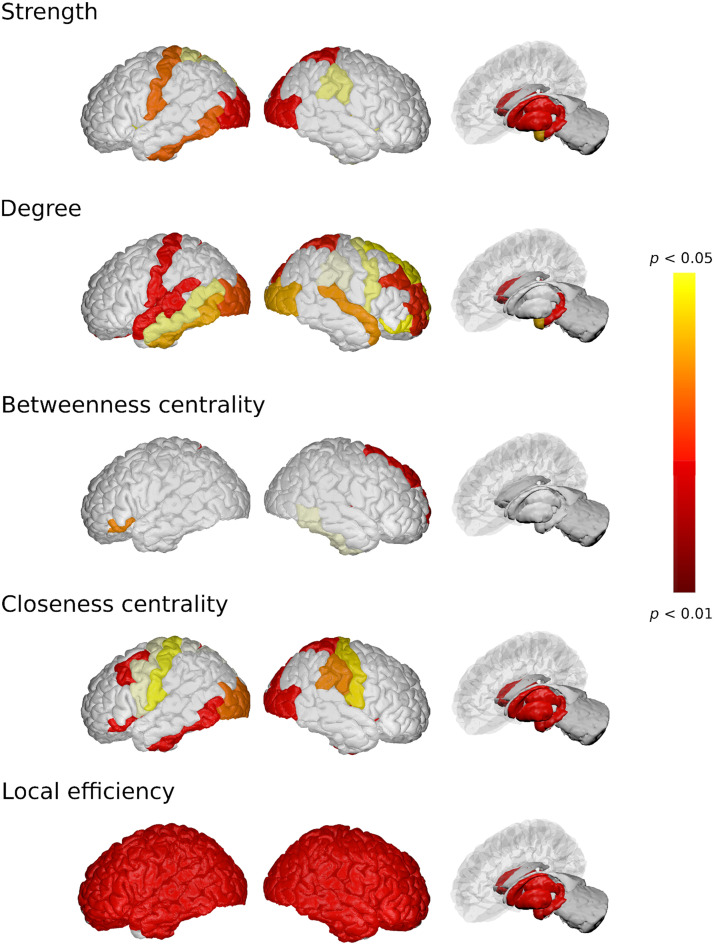
Depiction of statistically significant anatomical regions between people with MS and HVs using the graph theoretical descriptors obtained from the multilayer network analysis.

We analyzed a cohort of *n* = 125 people with relapsing-remitting MS (90 women), mean age of 45.66 ± 9.44 years, mean disease duration of 14.88 ± 8.07 years, and median EDSS of 2.0 (range 0–6.5), and a group of *n* = 18 HVs. The clinical and demographic from the final cohort are summarized in Table 1. Table 2 shows the number of regions identified with significant group differences (*p* < 0.05) in the network descriptors obtained from the multilayer network analysis. Details of these regions are depicted in Figure 3.

**Table 1.** Clinical and demographic data. Continuous variables are given as the mean ± standard deviation. EDSS = Expanded Disability Status Scale; MS = multiple sclerosis. *P* values obtained from comparing the groups.

	**Healthy volunteers (*n* = 18)**	**People with MS (*n* = 125)**	***p* value**
Age, years	36.62 ± 9.33	45.66 ± 9.44	0.001
Female, *n* (%)	15 (83%)	90 (66%)	<0.001
Disease duration, years	–	14.88 ± 8.07	–
Median EDSS score (range)	–	2.0 (0–6.5)	–

**Table 2.** Number of identified regions with significant differences (*p* < 0.05) in network descriptors obtained from the multilayer network analysis.

**Metric**	**No. of regions**	**No. of deep GM**	**No. of cortical GM**
Strength	31 / 76	12 / 14	19 / 62
Degree	31 / 76	7 / 14	24 / 62
Betweenness centrality	6 / 76	0 / 14	6 / 62
Closeness centrality	40 / 76	12 / 14	28 / 62
Local efficiency	76 / 76	14 / 14	62 / 62

Upon this setup, we validate our multilayer framework proposal by comparing the number of identified statistically different brain regions with respect to a randomized version of the multilayer structure, in which structural DTI links are shuffled. Results show that structural DTI data arranged as interlayer links is fundamental to identify more significant different regions between people with MS and HV (see Appendix 1 in the Supporting Information).

Most differences among the groups were observed in local efficiency measures, closeness centrality, node degree, and node strength (corrected *p* < 0.05). In all the nodes analyzed, local efficiency was lower in people with MS compared with the HVs (corrected *p* < 0.05), while closeness centrality and node degree were lower (corrected *p* < 0.05) in almost all deep GM regions. Table 3 shows the *p* values obtained from comparing HVs with people with MS in all deep GM regions. Regions involving left thalamus, left hippocampus, right thalamus, right caudate, and right accumbens were the regions with the largest differences between people with MS and HVs (corrected *p* < 0.05).

**Table 3.**
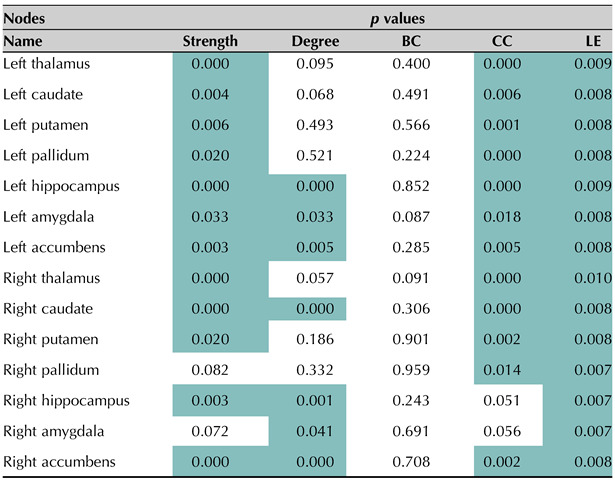
Detail of *p* values obtained from comparing healthy volunteers (HVs) with people with MS in all deep gray matter regions for each of the multilayer metrics: strength, degree, betweenness centrality (BC), closeness centrality (CC), and local efficiency (LE).

**Figure 4.**
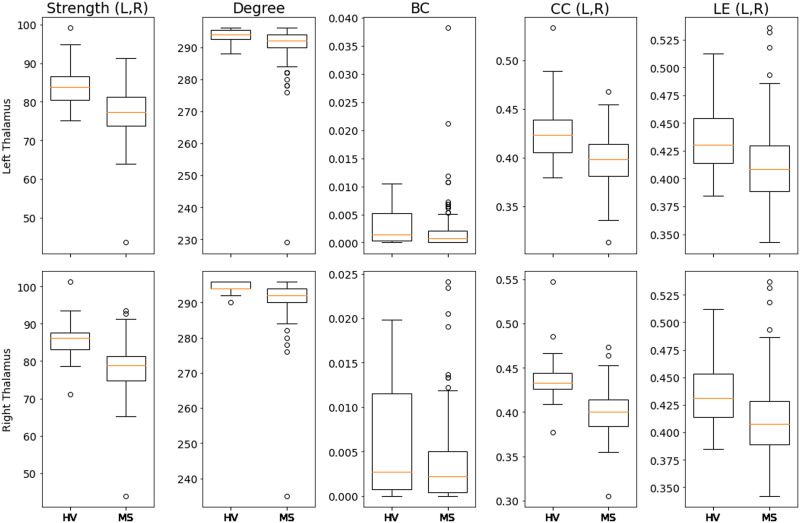
Boxplots comparing left and right thalamus of healthy volunteers (HV) and people with MS for each of the multilayer metrics: strength, degree, betweenness centrality (BC), closeness centrality (CC), and local efficiency (LE). For each metric, L and R stand for the statistical significance of the left and right thalamus, respectively.

Additionally, Figure 4 shows the boxplots comparing left and right thalamus for each multilayer descriptor for HVs and people with MS. According to the data presented in Table 3, we observe that the metrics local efficiency, closeness centrality, and degree best capture the differences between each group (see the Supporting Information for details and boxplots of all other regions).

We further demonstrate that the multilayer approach integrating morphological GM, structural DTI, and rs-fMRI functional networks may help to better understand the complex mechanisms underlying MS disease in comparison with other architectures (see Appendix 2 in the Supporting Information).

## DISCUSSION

This paper provides a multilayer approach by combining the information from different brain networks simultaneously. We computed the graph theoretical descriptors extracted by the combination of structural, functional, and morphological brain networks using well-established methodological approaches. To be able to merge the information between these three networks, we applied a common anatomical cortical atlas parcellation, and each individual region is represented by a node in our network (31 cortical labels and 7 subcortical regions per each brain hemisphere; Klein et al., 2017; Patenaude et al., 2011). This brain region distribution could be substituted by other atlas templates depicted by cytoarchitectonic or functional maps (Glasser et al., 2016). However, using this new multilayer approach, the same graph descriptors can still be computed through other atlases to explore other outcomes and interpretations.

The key to good multilayer architecture is combining each network efficiently. Multilayer architecture has two main features: the set of edges within a graph (between nodes of the same graph), and the set across graphs (between the same node in different graphs). Several approaches have been proposed to connect nodes between layers in a multilayer network: implicit (Battiston et al., 2017; Battiston et al., 2018) or explicit (Betzel et al., 2019; Guillon et al., 2017; Lim et al., 2019) connections between node replicas in the different layers, identity connections with varying weights (Betzel et al., 2019; Pedersen et al., 2018), or fully connected layer (Buldú & Porter, 2018; Ting et al., 2021) threshold restrictions. However, the definition is especially difficult when layers do not represent totally equivalent information (Damoiseaux & Greicius, 2009). Identity connections may restrict connectivity between layers, thus reducing the impact of large-range pathological changes of WM onto GM network properties or producing discordance (Lim et al., 2019). To overcome this challenging situation, our multilayer approach incorporates cross interaction between functional and morphological layers by using a structural connectivity factor that tries to mimic the WM topological organization and microstructural tissue properties within the multilayer architecture.

Another important step in our multilayer approach was to normalize the various networks, thus merging this information within a single multilayer architecture. As such, we normalized all values between 0 and 1 across different connectivity matrices to avoid one specific network or negative values being more influential than the others, which could lead to significant bias in network connectivity properties (Pedersen et al., 2018). Furthermore, statistical correction for common confounding variables at connectivity matrix level, such as age or gender, was applied in all the connectivity matrices to avoid bias (Solana et al., 2018).

GM morphological pipeline design produces matrices with very similar covariance patterns between different defined regions. Thus, it is difficult to merge the GM morphological similarity network with other networks, as GM network disruptions/morphological changes using graph theory metrics are less prominent than underlying pathological MS changes derived from diffusion and functional properties in mild disability patients (Zurita et al., 2018) with a median EDSS score of 2.0 (see Table 1). Overall, this study proves that using this multilayer approach to analyze the complex organization of the brain network could help identify abnormal patterns related to structural, morphological, and functional properties.

There is a loss of significance in different areas in HV and MS patients when comparing original and reshuffled data; this means that the nodes that remain significant after the reshuffling can rely also on functional and GM network contributions (see Appendix 1 in the Supporting Information) for their identification. Moreover, our findings show that a set of nodes are only significant under our proposed multilayer architecture, emphasizing the key role of DWI as interlink. Another key aspect of the proposed multilayer architecture is to analyze whether it provides better outcomes than other architectural combinations. Extra analysis has shown that the jointly morphological, structural, and functional brain networks provide better results than any other data combination in a classification task (see Appendix 2 in the Supporting Information).

As proof of concept, and to demonstrate its potential, our multilayer network approach was used to explore the hierarchical organization of brain loss in the context of neurological diseases such as MS. Findings point to deterioration of synchronized connectivity, which is particularly relevant for local efficiency and shows widespread loss across the brain. Moreover, most GM regions showed connectivity changes, particularly in strength and closeness centrality. The atrophy of deep GM areas occurs in early stages of MS, and extends to multimodal cortical regions over time (Eshaghi et al., 2018; Solana et al., 2021). In this context, we demonstrated that the network metrics applied to the new multilayer framework can pick up relevant network alterations in MS in most deep GM and in some cortical regions involved in visual areas (bilateral occipital and lingual areas) and cognition (Gabilondo et al., 2014; Riccitelli et al., 2020; Zhang et al., 2021). The frontal, temporal, and parietal lobes of MS patients are closely associated with new lesions in the first 10 years (Wybrecht et al., 2017). Furthermore, graph theory measures point out that the most atypical patterns in interconnected multilayer networks in MS stem from a weaker topological centrality (measure of degree and strength) and integrity (closeness centrality) measures, and increasing segregation (local efficiency) of node neighbors (Riccitelli et al., 2020; Zhang et al., 2021). However, the overall connectivity of the brain network in MS (betweenness centrality) patients is unchanged compared with HV, as reported previously (Llufriu et al., 2017).

We provide a scalable multilayer network architecture that merges more than two brain connectivity matrices in a single graph. This method enables researchers to examine multimodal MRI data in order to gain a better understanding of brain interactions in MS and other brain disorders, as well as a healthy brain. We expect that the set of graph metrics obtained from integrating connectivity matrices in a multilayer network will help detect subtle changes in brain organization as biomarkers of neurological and neuropsychiatric diseases, thus, opening the method to translation to the clinic or to clinical trials.

## LIMITATIONS

Our study undertakes several processing steps to obtain the final connectivity matrices and the multilayer network. Corrections for age and gender were applied, and the value scales and ranges changed. Although these steps were specifically chosen following the literature, others that would improve the sensitivity or specificity of the derived graph-mining metrics could be added. For example, the analysis performed in the morphological GM connectivity matrix to summarize the relationship between conjunctions of correlations of the cube in each brain region could be performed through other approaches (e.g., sum or mean).

Image processing in the DWI, fMRI, and T1 data could create controversy, as a large number of similar pipelines could be applied. Thus, we designed pipelines fully aligned to current state-of-the-art methods, without specifically tuning or optimizing any parameters for our dataset. Cerebellum has been excluded from the analysis because of the severe distortions at the level of brain stem region in DW images. Further research could focus on proper adjustment of the value range for each connectivity matrix, as this may impact graph connectivity metrics, such as considering the sign of the rs-fMRI instead of using the absolute values.

Another limitation of the study is the size of the MRI data used in the study. The single-center MS group was small, and the number of HVs was limited, with only 18 subjects with the three image modalities; therefore, further studies should be carried out on larger multicenter datasets to confirm our findings and expound their clinical effects. Despite data size, the findings are interesting and in line with other recently published MRI studies.

We selected the Desikan-Killiany-Tourville atlas based on an anatomical distribution. Other atlases are available, each with underlying meanings that will affect findings (Eickhoff et al., 2018). However, as discussed earlier, the multilayer architecture and the derived graph metrics can be computed using the approach presented, independent of the atlas chosen.

## CONCLUSIONS

This research presents a multilayer approach with corresponding graph metrics. This is the first time a multilayer approach merges morphological, structural, and functional connectivity information from the brain into an efficiently combined single network. Our multilayer approach was successfully applied to a cohort of people with MS, and interestingly, the proposed framework identified several brain regions showing synchronized connectivity deterioration. These encouraging results indicate that larger multicentric studies are warranted. Future work will incorporate more networks to the multilayer architecture and explore the potential of analyzing several networks simultaneously using graph neural networks.

## SUPPORTING INFORMATION

Supporting information for this article is available at https://doi.org/10.1162/netn_a_00258 and https://github.com/ADaS-Lab/Multilayer-MRI/.

## AUTHOR CONTRIBUTIONS

Jordi Casas-Roma: Conceptualization; Data curation; Formal analysis; Methodology; Validation; Writing – original draft; Writing – review & editing. Eloy Martinez-Heras: Conceptualization; Data curation; Formal analysis; Methodology; Validation; Writing – original draft; Writing – review & editing. Albert Solé-Ribalta: Conceptualization; Data curation; Formal analysis; Methodology; Validation; Writing – original draft; Writing – review & editing. Elisabeth Solana: Conceptualization; Data curation; Formal analysis; Investigation; Validation; Writing – review & editing. Elisabet Lopez-Soley: Conceptualization; Data curation; Writing – review & editing. Francesc Vivó: Conceptualization; Data curation; Writing – review & editing. Marcos Diaz-Hurtado: Conceptualization; Writing – review & editing. Salut Alba-Arbalat: Conceptualization; Data curation; Writing – review & editing. Maria Sepulveda: Conceptualization; Data curation; Writing – review & editing. Yolanda Blanco: Conceptualization; Data curation; Writing – review & editing. Albert Saiz: Conceptualization; Data curation; Writing – review & editing. Javier Borge-Holthoefer: Conceptualization; Formal analysis; Writing – review & editing. Sara Llufriu: Conceptualization; Data curation; Formal analysis; Methodology; Validation; Writing – review & editing. Ferran Prados: Conceptualization; Data curation; Formal analysis; Methodology; Validation; Writing – original draft; Writing – review & editing.

## FUNDING INFORMATION

Sara Llufriu, Instituto de Salud Carlos III (https://dx.doi.org/10.13039/501100004587), Award ID: PI15/00587. Albert Saiz, Instituto de Salud Carlos III (https://dx.doi.org/10.13039/501100004587), Award ID: PI18/01030. Albert Saiz, Red Española de Esclerosis Múltiple (https://dx.doi.org/10.13039/501100007747), Award ID: RD16/0015/0002. Sara Llufriu, Red Española de Esclerosis Múltiple (https://dx.doi.org/10.13039/501100007747), Award ID: RD16/0015/0003. Albert Saiz, Red Española de Esclerosis Múltiple (https://dx.doi.org/10.13039/501100007747), Award ID: RD12/0032/0002. Francesc Graus, Red Española de Esclerosis Múltiple (https://dx.doi.org/10.13039/501100007747), Award ID: RD12/0060/01-02.

## COMPETING INTERESTS

E. S. received travel reimbursement from Sanofi. E. L.-S. received travel reimbursement from Sanofi and ECTRIMS. M. S. received honoraria for speaking from Roche and Biogen, and travel reimbursement from Biogen, Sanofi, and Roche for national and international meetings. A. S. received consulting fees for serving on a scientific advisory board, speaking, and partaking in other activities for Merck-Serono, Sanofi, Biogen, Roche, TEVA, Novartis, Alexion, and Janssen. S. L. received consulting fees and honoraria for speaking from Biogen Idec, Novartis, TEVA, Genzyme, Sanofi, and Merck.

The author(s) disclose receipt of the following financial support for the research, authorship, and/or publication of this article. This work was funded by e-Health Center at Universitat Oberta de Catalunya, NIHR Biomedical Research Centre at University College London Hospitals NHS Foundation Trust and University College London, a Proyecto de Investigación en Salud (PI15/00587 to S. L. and A. S., and PI18/01030 to S. L. and A. S.), integrated into the Plan Estatal de Investigación Científica y Técnica de Innovación I+D+I, and cofunded by the Instituto de Salud Carlos III-Subdirección General de Evaluación and the Fondo Europeo de Desarrollo Regional (FEDER, “Otra manera de hacer Europa”); by the Red Española de Esclerosis Múltiple (REEM: RD16/0015/0002, RD16/0015/0003, RD12/0032/0002, RD12/0060/01-02); and by TEVA Spain, the Ayudas Merck de Investigación 2017 from the Fundación Merck Salud and the Proyecto Societat Catalana Neurologia 2017.

## TECHNICAL TERMS

Network: Also known as a graph, it is a set of entities (nodes) and relationships between pairs of nodes (links).
Multilayer network: Network composed of different layers, each representing a single type of relationship between nodes within one layer.
Node: Mathematical representation of an entity, also known as a vertex. In our case, it refers to a group of voxels.
Link: A mathematical representation of a relationship between two nodes, also called edge.
FA-weighted adjacency matrix: Square matrix where each cell (*i*, *j*) indicates the mean fractional anisotropy between node *i* and node *j*.
Adjacency matrix: Square matrix where each cell (*i*, *j*) indicates the link’s weight between node *i* and node *j*.
Gray matter morphological adjacency matrix: Square matrix where each cell (*i*, *j*) indicates the correlation of the GM patterns between node *i* and node *j*.
Gray matter functional adjacency matrix: Square matrix where each cell (*i*, *j*) indicates the signal synchronization between node *i* and node *j*.
Path: A path between two nodes is a list of links that connect the source and the target node.
Shortest path: Minimal path between two nodes, where the cost is defined as the sum of the links’ weight.
Ego network: Subgraph composed of a node and the set of nodes that are directly linked to it.

## Supporting Information

https://github.com/ADaS-Lab/Multilayer-MRI
