# Supplementary material for: Applying multilayer analysis to morphological, structural, and functional brain networks to identify relevant dysfunction patterns
Source: Netw Neurosci. 2022 Jul 1;6(3):916–33. doi: 10.1162/netn_a_00258 (PMC9810367; doi:10.1162/netn_a_00258)
Supplement: Supplementary file 1 [file netn-06-916-s001.pdf]

Casas-Roma, J., Martinez-Heras, E., Solé-Ribalta, A., Solana, E., Lopez-Soley, E., Vivó, F., Diaz-Hurtado, M., Alba-Arbalat, S., Sepulveda, M., Blanco, Y., Saiz, A., Borge-Holthoefer, J., Llufriu, S. & Prados, F. (2022). Supporting information for "Applying multilayer analysis to morphological, structural and functional brain networks to identify relevant dysfunction patterns." *Network Neuroscience*, 6(3), 916–933 . https://doi.org/10.1162/netn_a_00258

SUPPLEMENTARY MATERIALS

Applying multilayer analysis to morphological, structural and functional brain networks to identify relevant dysfunction patterns

Jordi Casas-Roma, Eloy Martinez-Heras, Albert Solé-Ribalta, Elisabeth Solana, Elisabet Lopez-Soley, Francesc Vivó, Marcos Diaz-Hurtado, Salut Alba-Arbalat, Maria Sepulveda, Yolanda Blanco, Albert Saiz, Javier Borge-Holthoefer, Sara Llufriu, Ferran Prados

Appendix 1. Randomization of the interlayer links

Our dataset contains two types of data regarding the cerebral tissue type into GM and WM: the first regarding the structural brain connectivity and the other morphological and functioning of the brain. We understand that these two types of data may be considered as different types of data in the multilayer architecture, especially since the centrality of a node in the multilayer is, in general, a non-trivial integration of the centralities obtained by the node replicas in the different layers considering the full multilayer approach. Thus, our objective was to work upon the structural networks but considering the mediation of the morphological and functional interactions. In this way, local centralities of node i in layer α will be influenced by local centralities nodes in layer β, but only of nodes that have been functionally related. This is indeed the role of the principal roles of the interlayer edges in multilayer networks. That being said, this is a generic definition and the particularities when considering the different centrality measures should be worked out.

In order to assess the influence of the structural DTI in the detection of nodes with significant differences on the centrality measures, we have performed an experiment randomising the DTI interlayer links. We have applied the process as follows: For each individual on the dataset (HV and people with MS), we took its interlayer links and we have randomised them (including weights), thus in this null model the number of links and the sum of strengths is maintained. We applied this to all brain regions and it showed a loss of significant different areas between HV and people with MS between original and reshuffled data (see Table below), keeping mainly deep grey matter areas as the most relevant (thanks to rsfMRI and GM contributions) as it would expect for MS disease (see Table S1).

Metric	Original data	Shuffled data
Strength	31/76	19/76
Degree	31/76	7/76
Betweenness centrality	6/76	4/76
Closeness centrality	40/76	17/76
Local efficiency	76/76	69/76

Table S1. *Number of identified regions with significant differences (p<0.05) in network descriptors obtained from the shuffled multilayer network analysis.*

To explore these results in depth, we have compared the results of Figure 4 of the main document with the results obtained with the shuffled network. Figure 4 displays the statistical significance of bilateral Thalamus, a major affected region with MS. However, even they do not show a large variation in the centrality measures across other different areas, all metrics have lost predictive power and have lower statistical significance to the point that the differences in the Right Thalamus are no more significant, as can be seen in Figure S1. See also the results in Table S2, where the randomised version of the multilayer structure identifies less significant regions than the original network, compared to Table 3 of the main document.

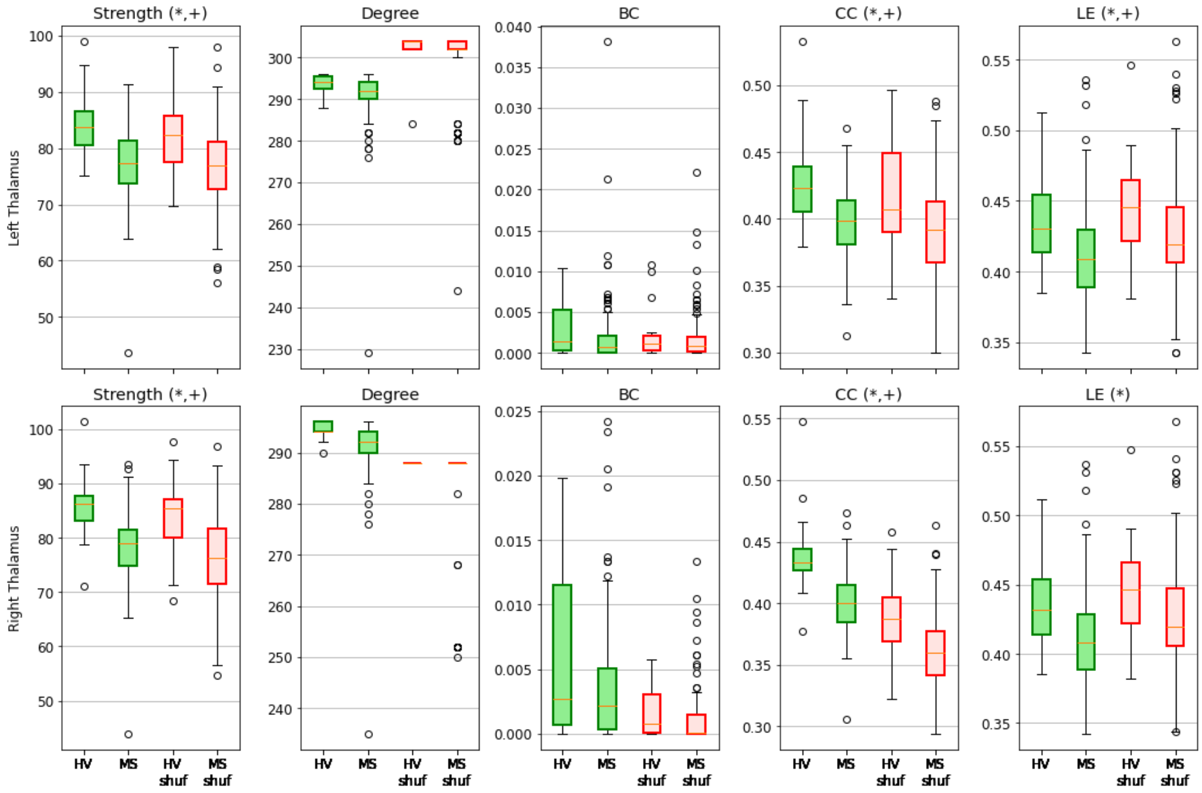

Figure S1. *Extended version of Figure 4 of the main document, where '*' and '+' stands for statistical significance of original and shuffled data, respectively.*

**Nodes**	***p*-values**				
**Name**	**Strength**	**Degree**	**BC**	**CC**	**LE**
Left Thalamus	0.306	0.248	0.569	0.011	0.048
Left Caudate	0.002	0.021	0.187	0.030	0.046
Left Putamen	0.003	0.404	0.359	0.004	0.050
Left Pallidum	0.024	0.343	0.954	0.260	0.044
Left Hippocampus	0.204	0.001	0.318	0.009	0.051
Left Amygdala	0.000	0.443	0.822	0.076	0.045
Left Accumbens	0.105	0.024	0.048	0.088	0.046
Right Thalamus	0.006	0.154	0.335	0.001	0.051
Right Caudate	0.001	0.132	0.540	0.026	0.048
Right Putamen	0.001	0.178	0.380	0.166	0.045
Right Pallidum	0.150	0.104	0.158	0.001	0.053
Right Hippocampus	0.046	0.150	0.029	0.075	0.046
Right Amygdala	0.165	0.288	0.199	0.088	0.047
Right Accumbens	0.352	0.101	0.511	0.082	0.045

Table S2. *Detail of p-values obtained from comparing healthy volunteers (HV) with people with MS in all deep grey matter regions for each of the multilayer metrics on the suffled network: Strength, Degree, Betweenness centrality (BC), Closeness centrality (CC) and Local efficiency (LE).*

Appendix 2. Classification task

In our previous paper (Solana et al. 2019) we used only structural DTI connectivity networks to train a classifier between people with MS and HV, and it showed that the results were suboptimal only using a single network. Based on that, we hypothesized that the impact of GM damage needs to be incorporated to improve classification performance. Upon our intuitions, we decided to move to an architecture that allows the morphological, structural and functional brain networks together, as the current work. In Table S3, we can see how the classification performance improves using jointly the morphological, structural and functional brain networks compared to other combinations thereof. We proceed as follows, we trained a Fully Connected Neural Network model taking as inputs the adjacency (or supra adjacency) matrices representing the brain of the subjects and producing two single outputs giving the probability of a subject being classified as HV or MS. We applied the process to the single layer representation and the different combination of two and three layers. The table also shows the accuracy (mean and standard deviation) obtained applying a stratified k-fold cross-validation (k=10) experiment:

	**AUC ROC**	**AUC Precision-Recall**	**Accuracy**
**DTI**	0.62 ± 0.03	0.93 ± 0.10	0.89 ± 0.02
**GM**	0.54 ± 0.02	0.92 ± 0.01	0.89 ± 0.00
**RS-fMRI**	0.61 ± 0.03	0.92 ± 0.01	0.89 ± 0.00
**DTI + GM**	0.62 ± 0.03	0.93 ± 0.01	0.89 ± 0.00
**DTI + RS-fMRI**	0.65 ± 0.05	0.94 ± 0.01	0.89 ± 0.00
**GM + RS-fMRI**	0.65 ± 0.03	0.93 ± 0.01	0.89 ± 0.00
**DTI + GM + RS-fMRI**	0.68 ± 0.06	0.94 ± 0.01	0.90 ± 0.01

Table S3. *Detail of accuracy (mean and standard deviation) from classification task of the single layer representation and the different combination of two and three layers*

The results of this experiment shows that the combination of the multiple types of data helps in the classification process, showing the capabilities of our modelling approach (2 layers, with structural DTI as interlink) surpass any other combination.
